# Orbital Lymphoma Masquerading as Euthyroid Orbitopathy

**DOI:** 10.7759/cureus.34885

**Published:** 2023-02-12

**Authors:** Muhammad Waqar Sharif, Sai Mungara, Kelash Bajaj, Pablo Amador, Nuvneet Khandelwal

**Affiliations:** 1 Internal Medicine, Texas Tech University Health Science Center at Permian Basin, Odessa, USA; 2 Hematology and Oncology, Texas Tech University Health Science Center at Permian Basin, Odessa, USA; 3 Hospital Medicine, Queens North Hawaii Community Hospital, Waimea, USA

**Keywords:** bilateral proptosis, euthyroid orbitopathy, primary orbital lymphoma, mucosa-associated lymphoid tissue (malt) lymphoma, thyroid-associated orbitopathy

## Abstract

Thyroid eye disease (TED), also called Graves orbitopathy (GO), is the most common diagnosis of orbital tissue inflammation. It is typically associated with the onset of hyperthyroidism, an autoimmune response to excess amounts of thyroid hormone. However, a visible and palpable lump, strictly unilateral or gross asymmetric eye involvement, non-axial (eccentric) proptosis, a lack of lid retraction or lid lag on downward gaze, or weakened muscle function (suggestive of tendon involvement) are the key features of non-TED mediated ocular involvement, as was found in the case we report here. Orbital lymphoma should always be suspected and excluded in all cases of orbital inflammation. Our patient was diagnosed with mucosa-associated lymphoid tissue (MALT) lymphoma at 27 years of age, two years after the diagnosis of euthyroid ophthalmopathy. This case highlights the need to include space-occupying lesions in the differential diagnosis of proptosis and gaze restrictions, even in younger patients.

## Introduction

Graves orbitopathy (GO) is one of the leading causes of inflammation of orbital and periorbital tissue, accounting for around 60% of all orbital pathologies in the middle-aged population [1]. Graves disease accounts for 25-30% of all ophthalmopathy cases, but 1-2% of ophthalmopathy patients do not exhibit any overt thyroid dysfunction [2]. Between 5 and 10% of orbitopathies can be attributed to neoplasms, half of which are lymphomas. and 40% of orbital lymphomas derive from mucosa-associated lymphoid tissue (MALT). The presentation is bilateral in 25% of cases [3]. Around 40% of patients have systemic involvement at the time of the diagnosis, whereas 60% have a localized form, which usually develops into a systemic lymphoma within five years [4]. Therefore, orbital lymphoma should always be suspected and excluded in all cases of orbital inflammation. We present a case of a young patient initially diagnosed with euthyroid ophthalmopathy and later found to have orbital MALT lymphoma.

This article was previously presented as a poster at the Annual Research Day at Texas Tech University Health Sciences Center, Permian Basin on May 6, 2022.

## Case presentation

A 27-year-old female with uncontrolled type II diabetes mellitus, endometriosis, polycystic ovary syndrome (PCOS), and euthyroid ophthalmopathy diagnosed in 2019, who had been legally blind in the left eye since 2021, presented with a worsening blurring of the vision in the right eye. Ophthalmic examination showed bilateral proptosis, conjunctival chemosis, and deviation of the gaze in the right eye. The rest of the examination was unremarkable.

The patient had presented with ophthalmopathy in 2019. Due to classic ocular features of GO, normal thyroid function tests and thyroid-stimulating hormone (TSH), including serological tests like anti-TSHR, thyroid peroxidase antibody, thyroid-stimulating antibody, and thyroglobulin antibodies, the diagnosis of euthyroid orbitopathy had been made at that time. Results of an orbital MRI in 2019 had shown a bilateral enlargement of the medial recti muscles compressing both the optic nerves and orbital fat edema. Her proptosis, eye swelling, and visual acuity had improved by the second day of steroid administration. The patient had been discharged on oral steroids and immunotherapy, targeting thyroid orbitopathy with teprotumumab. 

After the initial improvement over the past two years, she had been lost to follow-up in the clinic, and eventually, her visual acuity started deteriorating. At the start of 2021, she was declared legally blind in her left eye. She presented to us with the blurring of the vision in the right eye. In October 2021, a biopsy of the left rectus muscle was done, which came out to be positive for extranodal marginal zone B-cell lymphoma (mild-type) (MALT lymphoma) with plasmacytic differentiation associated with increased IgG4 (plasma cells average: 82% IgG4/IgG). Plasma IgG4 levels were normal. There was fibroconnective tissue with the striated muscle mainly infiltrated and dissected by lymphocytic infiltrate, with a few plasma cells and rare histiocytes. We started the patient on R-CHOP (rituximab, cyclophosphamide, doxorubicin hydrochloride, Oncovin, and prednisone) chemotherapy; her blurring of the vision in the right got better, and in the interim, we also started her on cytarabine intrathecal chemotherapy for better penetration. During the second outpatient intrathecal cytarabine chemotherapy session, the patient had two witnessed tonic-clonic seizure episodes and was admitted to the hospital for further evaluation. 

**Figure 1:**
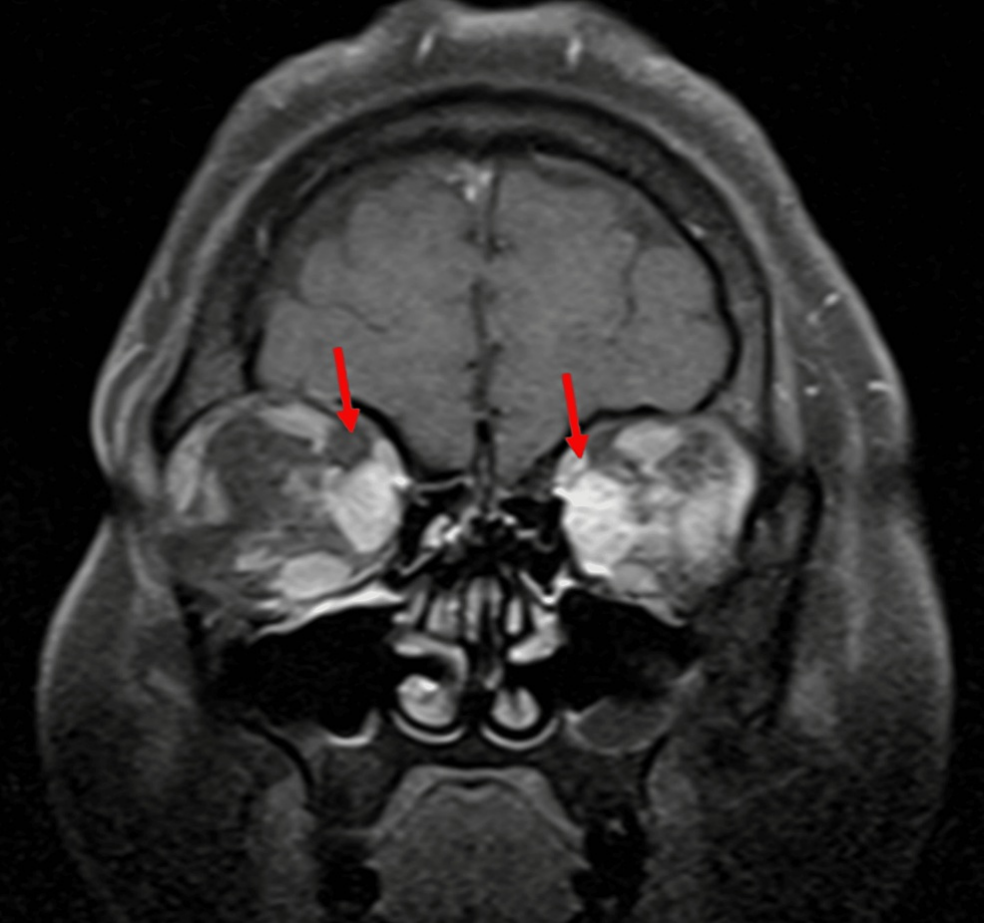
Orbital MRI (T1, coronal view) showing significant enlargement of extraocular muscles (bilateral medial recti indicated by red arrows) MRI: magnetic resonance imaging

**Figure 2:**
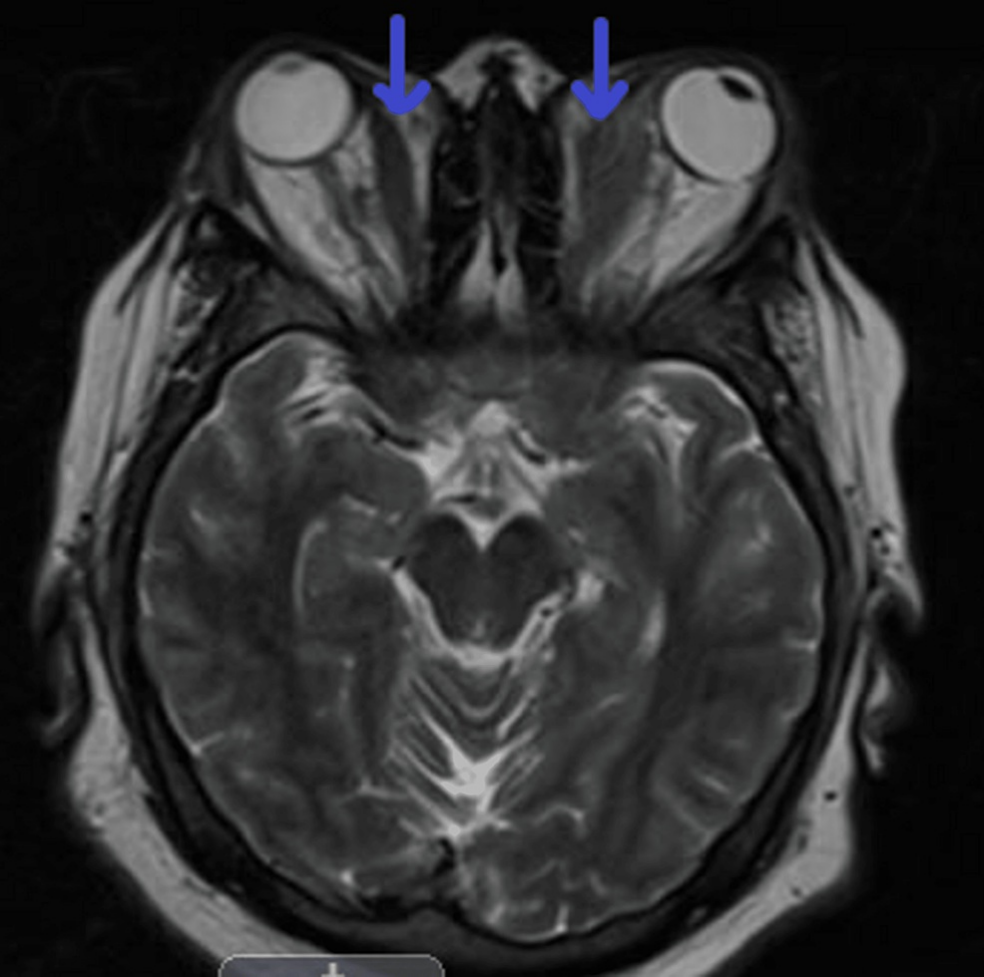
Orbital MRI (T2, axial view) showing significant enlargement of extraocular muscles (bilateral medial recti indicated by blue arrows) MRI: magnetic resonance imaging

A CT scan was done, which revealed a fusiform conal mass infiltrating the left rectus muscle and wrapping around the optic nerve sheaths; later, MRI revealed the same (Figures 1, 2). In addition, we found less severe diffuse fusiform enlargement of the right medial rectus muscle with a cluster of extra coronal nodules wrapping around the scleral insertion of the medial rectus muscle and partially extruding into the conjunctiva along with B/L severe exophthalmos.

A lumbar puncture for CSF fluid analysis and flow cytometry was negative for malignant infiltration. In addition, CT imaging of the chest, abdomen, and pelvis was also negative for any metastasis. The patient was observed in the hospital for 24 hours. She had no episodes of seizures and had a clear clinical improvement in vision in the right eye. She was discharged home with levetiracetam 500 mg BID with a provisional diagnosis of seizures 2/2 meningeal irritation from intrathecal cytarabine infusion. She underwent six chemotherapy sessions post-discharge, resulting in significant clinical and radiological improvement.

**Figure 3:**
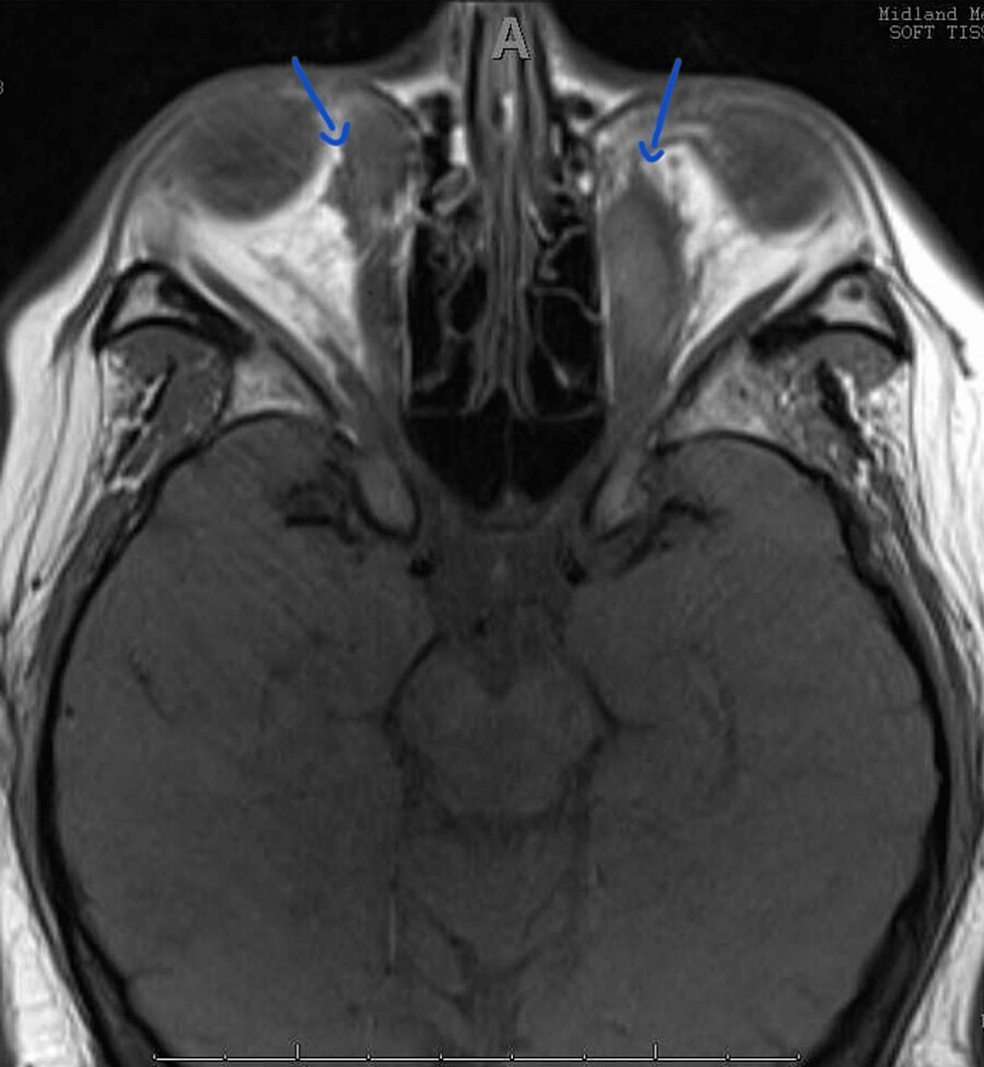
Post-treatment orbital MRI (axial view) showing significant reduction in enlargement of extraocular muscles (bilateral medial recti indicated by blue arrows) MRI: magnetic resonance imaging

Post-treatment MRI revealed a mass-like enlargement of the bilateral medial and inferior rectus muscles with a mildly irregular contour. Equivocal involvement of the superior oblique muscle was seen. The findings improved compared to the prior exams, as shown in Figure 3. There was thinning versus stretching of the right more than the left optic nerves. The right intraorbital optic nerve demonstrated T2 prolongation. The previously noted encasement of the left optic nerve was resolved, leading to mild improvement in left eye vision.

## Discussion

Non-Hodgkin’s lymphoma (NHL) is the sixth most common malignancy in the USA among females and seventh among males. According to the American Cancer Society, there were 80,470 cases and 20,250 deaths due to NHL in 2022 [5]. NHL can arise from nodal or extranodal sites. Primary ophthalmic lymphoma constitutes 1-2% of all NHLs and 5-15% of all extranodal NHLs [6,7]. Ophthalmic NHL is mostly of B-cell origin and can be in ocular adnexal regions (orbit, conjunctiva, lacrimal gland, or eyelid) or intraocular (cornea, retina, choroid, or ciliary body), which are considered a subset of CNS lymphoma [8,9].

The incidence of thyroid eye disease (TED) is 2.9 and 16.0 cases per 10,000 population per year among males and females, respectively. The higher prevalence in females is associated with a higher incidence of hyperthyroidism in them [10]. Unilateral TED is seen in nearly 4-14% of cases. However, a visible and palpable lump, strictly unilateral or gross asymmetric eye involvement, non-axial (eccentric) proptosis, a lack of lid retraction or lid lag on downward gaze, or weakened muscle function (suggestive of tendon involvement) are the critical features of non-TED mediated ocular involvement, as in our patient [11]. Initially, our patient was diagnosed with euthyroid ophthalmopathy and was treated with prednisone and teprotumumab; proptosis significantly decreased from 30 mm to 20 mm on discharge. The OPTIC-X study has shown that teprotumumab, administered once every three weeks, effectively reduced proptosis and diplopia [12]. Our patient, after an initial improvement in vision, diplopia, and proptosis, deteriorated, following which a biopsy was done, which revealed MALT lymphoma. According to a report, the median latency between the diagnosis of thyroid orbitopathy and the development of periocular lymphoma in patients was 17.5 years [13]. According to another case report, the time to diagnosis was five years [14]. Our patient was diagnosed with lymphoma within two years of the diagnosis of euthyroid ophthalmopathy.

Among a cohort of ocular adnexal lymphoma patients, the proportion of MALT lymphoma ranged from 50 to 87%. In a recently published report from Korea, 87% of all orbital and ocular adnexal lymphoma cases were MALT lymphoma [15]. The mechanism of the development of lymphoma in thyroid ophthalmopathy is ascribed to chronic lymphocytic stimulation. The pathogenesis of TED involves autoimmunity against the TSHR, primarily present on the orbital fibroblasts and adipocytes, which acts as the primary autoantigen. Unilateral marginal zone B-cell NHL and MALT-lymphoma have been reported in several cases [16,17,18].

Whole body CT, cerebral MRI, and lumbar puncture were negative for secondary localization of lymphoma in our patient. Thus, the disease was classified as stage IEA according to the Ann Arbor classification and T3N0M0 according to the TNM classification. Our patient was started on intrathecal R-CHOP chemotherapy. A few studies have demonstrated that radiotherapy alone is better for low-grade lymphoma, but chemotherapy with or without radiotherapy has shown better results than radiotherapy alone for aggressive disease. The R-CHOP regimen has proven to be efficacious in treating extranodal marginal zone lymphoma compared to other chemotherapy regimens [19]. Neurotoxicity with intrathecal R-CHOP has been reported [20], and our patient had tonic-clonic seizures during her second outpatient intrathecal cytarabine chemotherapy session.

## Conclusions

The most frequent cause of bilateral proptosis is GO. Between 5 and 10% of orbitopathy can be attributed to neoplasms, half of which are lymphomas. This case emphasizes the need to include space-occupying lesions in the differential diagnosis of proptosis and gaze restrictions. Physicians should be mindful that bilateral thyroid ophthalmopathy, although uncommon, may mimic orbital lymphoma.
